# Mitochondria operate as signaling platforms in yeast aging

**DOI:** 10.18632/aging.100914

**Published:** 2016-02-27

**Authors:** Younes Medkour, Vladimir I. Titorenko

**Affiliations:** Department of Biology, Concordia University, Montreal, Quebec H4B 1R6, Canada

**Keywords:** aging, yeast, geroprotective pharmaceuticals, mitochondrial lipidome and proteome, mitochondria as signaling organelles in aging

Mitochondria are vital to physiology and health of eukaryotic organisms across phyla. These organelles generate the majority of cellular ATP and create biosynthetic intermediates for amino acids, nucleotides and lipids [1]. Mitochondria can also operate as signaling platforms and structural scaffolds for coordinating diverse cellular responses to changes in a variety of physiological conditions [2, 3]. Therefore, the functional state of mitochondria is crucial for a plethora of cellular processes, including cell growth, division, differentiation, homeostasis, metabolism, stress response, signaling, immune response, survival and death [1 - 4]. Because mitochondrial functionality gradually declines with age in evolutionarily distant organisms, such age-related deterioration of mitochondrial function is regarded as the universal hallmark of cellular and organismal aging [5]. The budding yeast *Saccharomyces cerevisiae*, a unicellular eukaryote, has been intensively used as a model organism for uncovering mechanisms linking mitochondrial functionality and cellular aging [3].

Our recent studies have revealed how a geroprotective chemical compound delays yeast chronological aging by causing an age-related remodeling of mitochondrial lipidome [6], how such remodeling elicits changes to mitochondrial morphology and functionality [6], and how such changes enable mitochondria to operate as signaling platforms that exploit a distinct set of transcription factors to choreograph a longevity-extending transcriptional program for many nuclear genes [7]. We found that exogenously added lithocholic bile acid (LCA), which is known to slow the chronological aging of *S. cerevisiae*, enters yeast cell, amasses within a double membrane delimiting mitochondria and resides primarily in the inner mitochondrial membrane [6]. After being confined to mitochondrial membranes, LCA elicits specific changes in the concentrations of mitochondrial membrane phospholipids; these changes occur in an age-related fashion and are believed to be due to a characteristic remodeling of pathways for phospholipid synthesis and movement within both mitochondrial membranes [6]. The resulting major changes in mitochondrial membrane lipidome cause a substantial enlargement of mitochondria, decrease the number of these organelles, significantly increase the total length on mitochondrial cristae, and reduce the extent of connectivity between these cristae and the inner mitochondrial membrane [6]. These extensive alterations in mitochondrial abundance and morphology incite a distinct pattern of changes in the age-related chronology of such key longevity-defining processes as mitochondrial respiration, membrane potential maintenance, ATP synthesis and reactive oxygen species homeostasis [6]. Such specific changes to the age-related chronology of mitochondrial functionality allow mitochondria to function as signaling platforms that coordinate a stepwise establishment of a distinct transcriptional program for many nuclear genes [7]. The observed age-related rewiring of transcriptional patterns is choreographed by a distinct set of transcription factors [7]. These transcription factors respond to different aspects of altered mitochondrial functionality in yeast cells exposed to LCA; they include the following regulators of transcription: 1) the Rtg1, Rtg2 and Rtg3 protein components of the mitochondrial retrograde signaling pathway; 2) the Sfp1 protein component of the mitochondrial back-signaling pathway; 3) the Aft1 transcription factor modulated by the concentrations of iron-sulfur clusters, which function as inorganic cofactors of numerous mitochondrial, nuclear and cytosolic proteins; 4) a transcription factor involved in the unfolded protein response pathway of mitochondria-to-nucleus communications; this pathway is elicited in response to reduced concentrations of the mitochondrial protease Yme1 and the mitochondrial peptide exporter Mdl1; and 5) the transcription factors Yap1, Msn2/Msn4, Skn7 and Hog1, all modulated by mitochondrially generated reactive oxygen species [7]. Based on these observations, we proposed a model for how LCA-driven changes in mitochondrial proteome and functionality early and late in life of chronologically aging yeast coordinate a stepwise development of an aging-delaying cellular pattern and its maintenance throughout lifespan [7].

In sum, we demonstrated that the geroprotective chemical compound LCA can delay the onset and slow the progression of chronological aging in yeast by causing an age-related remodeling of mitochondrial lipidome. Our findings revealed that such remodeling of mitochondrial lipidome alters mitochondrial morphology and functionality, thereby enabling mito-chondria to act as signaling platforms that can 1) choreograph a longevity-extending transcriptional program for many nuclear genes governed by a distinct set of transcription factors; and 2) orchestrate a gradual establishment and long-term maintenance of a longevity-extending cellular pattern. Thus, targeting the discovered role of mitochondria as signaling platforms in yeast aging has potential as a novel therapeutic strategy for slowing aging, improving health, attenuating age-related pathologies and delaying the onset of age-related diseases in humans.
